# Diseases Admitted under the Care of the Physicians of the Westminster Hospital, from the 20th of August, to the 24th of September, 1800

**Published:** 1800-11

**Authors:** 


					474 State of Diseases in London.
J)ifeafes 'admitted under the Care of the Phyjicians of the Wejlminfter
Hofpital, from the 20th of Augujl, to the zi^th of September, 1800.
Continued Fever - - - 23
Intermittent - - - - - 1
Sore Throat ----- 2
Splenitis - - - - - - 1
Amenorrhea - - - 6
Anafarca - - - - -3
Afcites ------ 2
Afthenia ----- 4
CephalEca ----- 3
Chlorofis - - - - - 3
Cholera ------ 7
Colic l
Cough ------ 5
Diarrhoea - - - - -4
Dyfentery - I
Dyfpepfia ----- 3
Dyfpncea ------ 1
Enterodynia ----- 4
Erythema ----- i
Gaftrodynia ----- 5
Haemoptyfis ----- 1
Hooping Cough - - - - 1 ?
Itch - -- -- *-'2
Jaundice - - - 1
Leucorrhcpa ? - -? - - -I '
Lumbago ------ 1
Menorrhagia - - - - 1
Paralyfis ------ I
Pleurodynia v - - - - . i
Somnambulifmus - - - 1
Rheumatifm - - - 5
Sciatica - - - 1
Struma - . - - -. - - 2
Urticaria - - - - - - I
Tinea - - - - -? - 1 ?
Vomiting - - - - - 1
1

				

